# Different Antiplatelet Strategies for Radial Artery Protection After Transradial Coronary Angiography—A Prospective Observational Cohort Study

**DOI:** 10.3389/fcvm.2022.913008

**Published:** 2022-06-14

**Authors:** Zheng Qin, Xingsheng Yang, Wanjun Cheng, Jianlong Wang, Zening Jin

**Affiliations:** ^1^Department of Cardiovascular, Beijing Tiantan Hospital, Capital Medical University, Beijing, China; ^2^Department of Cardiology, Beijing Anzhen Hospital, Capital Medical University, Beijing, China

**Keywords:** radial artery occlusion, transradial access, single-antiplatelet therapy, dual-antiplatelet therapy, coronary angiography

## Abstract

**Introduction:**

Radial artery occlusion (RAO) after transradial access is a common thrombotic complication. A meta-analysis has proven that RAO incidence in transradial coronary angiography (TRCA) settings was significantly higher than that in percutaneous coronary intervention settings. This prospective observational cohort aimed to evaluate radial artery protection after TRCA with different antiplatelet strategies.

**Methods:**

A total of 2,316 patients undergoing TRCA was enrolled and divided into two groups: single-antiplatelet and dual-antiplatelet groups. Radial artery patency was evaluated by ultrasound before, at 24 h, and 30 days after TRCA. The primary endpoint was RAO incidence at 30 days after TRCA.

**Results:**

A total of 66 RAO was found on ultrasonography at 30-day follow-up (incidence: 2.8%). In the dual-antiplatelet group, the rate of RAO was significantly lower compared with the single-antiplatelet group (1.8 vs. 4.0%; odds ratio (OR): 0.41; 95% confidence interval (CI): 0.24–0.70; *p* = 0.001). The rate of self-recanalization in the dual-antiplatelet group was significantly higher than that in the single-antiplatelet group (73.68 vs. 44.12%, *p* < 0.001). However, there was no statistical difference in delayed occlusion of radial artery between the two groups (0.5 vs. 0.2%, *p* = 0.140). Unexpectedly, this study also showed no significant difference in bleeding risk between the groups.

**Conclusion:**

Dual-antiplatelet therapy for 1 month after TRCA was associated with a reduced risk of RAO and deemed safe.

## Introduction

Since transradial intervention (TRI) was first introduced by Campeau (1), transradial access (TRA) has become the preferred route for coronary angiography (CA) and percutaneous coronary intervention (PCI). This has been mainly driven not only by the reduction in access site-related complications (2) and in costs (3) and an increase in patient comfort (4), accounting for more than 50% in Europe, Asia, and the United States (5), but also by the reduced mortality in high-risk patients who present with an acute coronary syndrome (ACS) (6). Therefore, the European Society of Cardiology (7) and American Heart Association (8) recommend TRA as a preferred approach for ACS patients undergoing invasive management. Radial artery occlusion (RAO) after TRA is a common structural complication (9). It prohibits the reuse of the same artery for future transradial coronary procedures and as a graft for coronary artery bypass surgery (CABG).

The process of TRA may lead to endothelial trauma and endothelial dysfunction manifesting as thrombosis (10, 11), a process that may last a week or more (12). A meta-analysis involving 66 studies with 31,345 participants has confirmed that RAO incidence during TRCA was significantly higher at 8.8% compared to 4.5% during PCI (*p* < 0.001) (9). This difference might be related to intraoperative heparin dosage and long-term antiplatelet therapy. A recently published systematic review (13) demonstrated that anticoagulation during TRA can reduce early RAO (8.0%; 95% CI, 6.1–10.6 vs. 4.4%; 95% CI, 3.5–5.5; *Q* = 10.69; *p* = 0.001), but no significant benefit was observed for late RAO (5.4%; 95% CI, 3.7–7.8 vs. 5.0%; 95% CI, 3.6–6.8; *Q* = 0.11; *p* = 0.745), indicating that short-term intraoperative heparin application cannot provide long-term benefits. Therefore, long-term antithrombotic treatment after an operation is necessary. A subgroup analysis of this study (13) showed that, even with high doses of heparin, the RAO% in patients undergoing TRCA was higher than that in patients undergoing PCI, also suggesting that antiplatelet therapy might be associated with RAO. However, there are no current studies on different antiplatelet strategies for radial artery protection in patients after TRCA.

## Materials and Methods

### Method and Population

This prospective observational cohort study was conducted at Beijing Tiantan Hospital and Beijing Anzhen Hospital. Additionally, the specification of the intended analyses was determined before conducting this study. In this study, all patients were given dual-antiplatelet therapy (aspirin 100 mg/d + clopidogrel 75 mg/d) before surgery. A total of 5,068 patients was assessed for eligibility in the study from December 2018 to May 2020. In the present study, the inclusion criteria were as follows: (1) Age of more than 18 years and (2) ultrasound examination of radial artery performed before operation. Additionally, we used the following exclusion criteria: (1) Undergoing *ad hoc* PCI; (2) perioperative death; (3) no tolerance to aspirin, clopidogrel, and heparin; (4) a history of previous ipsilateral TRA; (5) failed radial artery puncture; (6) preoperative ultrasonography that indicated abnormal anatomical structure or occlusion of the radial artery; (7) other complications, such as serious liver diseases, long-term dialysis, need for anticoagulation therapy, bleeding/coagulation dysfunction, scleroderma, and other immune diseases; and (8) systolic pressure < 100 mm Hg. Lumen occlusion was defined as the absence of blood flow through the radial artery. In the end, 2,373 patients met the above-mentioned criteria and were enrolled in this study. In the present study, we divided the patients into single-antiplatelet (aspirin 100 mg/d) and dual-antiplatelet groups (aspirin 100 mg/d + clopidogrel 75 mg/d) mostly according to angiographic evaluation results. Meanwhile, we also considered age, medical history, and patient’ will.

**FIGURE 1:**
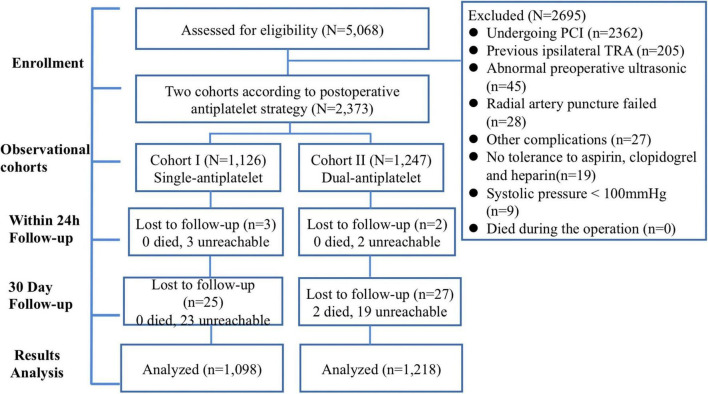
Flowchart of study. PCI, percutaneous coronary intervention; TRA, transradial access.

All patients were followed up in the hospital. Patients were followed up with a color Doppler ultrasound within 24 h and 30 days after the operation. Thus, 2,368 patients were successfully followed up for the first time, with 5 patients missing. At the 30-day follow-up, 2,316 patients were included in the final statistical analysis. A flowchart of the study is shown in Figure 1. The Clinical Research Ethics Committee of Beijing Tiantan Hospital and Beijing Anzhen Hospital, Capital Medical University (2018032X), approved this study, and all patients provided written informed consent for participation in this study.

### Transradial Access and Hemostasis

#### Transradial Access and Procedure

Under the premise of a sterile environment and sufficient anesthesia, a puncture was made at the strongest point of palpation of the radial artery with a Teflon-sheathed needle. Once continuous blood flow was observed, the guidewire was immediately moved forward through the needle. The 6-French and 16-cm hydrophilic coated interventional sheath (RADIFOCUS INTRODUCER II, Terumo company, Tokyo, Japan) was inserted into the radial artery alongside the guidewire to complete the puncture and catheterization process. 5,000 IU of unfractionated heparin was administered immediately after the sheath tube was inserted into the radial artery. Diagnostic coronary angiography was performed with the 5 Fr catheter, and the use of other devices was left at the surgeon’s discretion.

#### Hemostasis

After coronary angiography, the sheath tube was removed. At the radial artery puncture site, we used the artery compression tourniquet with the necessary pressure (slowly releasing the pressure until the puncture point bled and then applying minimal pressure until the bleeding stopped) for hemostasis. At last, we determined the radial pulse at the proximal end of the hemostatic device. To achieve the shortest hemostasis time, we attempted to remove the hemostat every 2 h after 2 h of hemostasis. All patients were referred to achieve the shortest hemostasis time; thus, we tried to remove the hemostatic device every 2 h when the compression lasted more than 2 h. Patency hemostasis was not applied to the entire population, intending to demonstrate a real-world hemostasis regimen.

### Evaluation of Radial Artery Patency: Ultrasound

All participants underwent ultrasound measurements of the radial artery before, within 24 h, and 30 days after the TRCA procedure. In the present study, the ultrasonography measurements were all performed by 2 independent and experienced operators who were blinded to the study groups, using an Aplio 500 ultrasound system (Toshiba, Japan) with 4–9 MHz linear array transducers. If a disagreement occurred, we solved it by a discussion with a senior researcher. The participant had a supine rest for 10 min before measurement, followed by right radial artery parameters measurement at 3 sites of the radial artery: puncture point, distal sheath catheter, and the midpoint. The 6 parameters measured at these 3 positions were measured by two-dimensional ultrasound and Doppler ultrasound, including intima-media thickness (IMT), inner diameter (ID), and pulsatility index (PI).

### Study Endpoints

In this study, the primary endpoint was RAO at 30 days after angiography. The secondary endpoints included self-recanalization (occlusion within 24 h but recanalization at 30 days after angiography), delayed occlusion (occlusion occurring (> 24 h) after angiography), and Bleeding Academic Research Consortium (BARC) type 2–5. The RAO was judged by the visible occlusion on two-dimensional ultrasound and the disappearance of the Doppler flow signal at the original entrance position. To document the bleeding events, the participants were contacted by cell phone at 7, 14, and 30 days after angiography.

### Statistical Analysis

Continuous variables of normal distribution were expressed as mean ± standard deviation, and the difference was expressed as the *t*-test of two independent samples. Non-normal distribution was expressed as median/quartile, and the difference was expressed as a rank-sum test. The categorical variables were expressed as percentages, and the difference was expressed as Chi-square test. The rate of RAO was calculated as the number of patients with confirmed RAO by ultrasound. Multivariate logistic regression models were established to analyze independent risk factors associated with the RAO at 30 days after the procedure. Statistical significance was accepted at the 95% confidence level (CI) (two-sided *p* ≤ 0.05). SPSS software for Windows (version 24.0, SPSS Inc., Chicago, Illinois) was used for statistical analyses.

## Results

### Baseline Demographic, Ultrasonic, and Operation Process Characteristics

The baseline demographic and clinical characteristics are demonstrated in Table 1. A total of 2,316 patients was included in the statistical analysis, divided into 1,098 patients in the single-antiplatelet group and 1,218 patients in the dual-antiplatelet group. The average age of the population was 61.13 ± 9.51, and 23.1% were women. There were no significant differences between the two groups in terms of demographic data, previous medical history, and medical treatment.

**TABLE 1:** Baseline demographic and clinical characteristics.

	Overall (*n* = 2,316)	SAPT (*n* = 1,098)	DAPT (*n* = 1,218)	*p*-value
Age, year	61.13 ± 9.51	60.96 ± 9.39	61.28 ± 9.62	0.418
Male, n (%)	1,780 (76.9)	828 (75.4)	952 (78.2)	0.117
BMI, kg/m2	26.43 ± 2.38	26.51 ± 2.46	26.36 ± 2.31	0.133
SBP, mmHg	130.62 ± 16.01	130.91 ± 16.07	130.36 ± 15.96	0.411
DBP, mmHg	76.43 ± 10.19	76.18 ± 10.29	76.65 ± 10.10	0.269
Smoking, n (%)	1,184 (51.1)	543 (49.5)	641 (52.6)	0.127
Drinking, n (%)	454 (19.6)	219 (19.9)	235 (19.3)	0.693
**History, n (%)**				
Diabetes mellitus	614 (26.5)	280 (25.5)	334 (27.4)	0.296
Hypertension	1,538 (66.4)	730 (66.5)	808 (66.3)	0.941
Hyperlipidemia	1,160 (50.1)	538 (49.0)	622 (51.1)	0.32
Prior stroke	112 (4.8)	55 (5.0)	57 (4.7)	0.712
**Laboratory evaluation**				
TG, mmol/L	1.51 (1.09, 2.15)	1.51 (1.10, 2.14)	1.52 (1.08, 2.16)	0.799
TC, mmol/L	12.12 ± 4.18	4.18 ± 1.14	4.18 ± 1.16	0.935
LDL-C, mmol/L	7.38 ± 2.49	2.50 ± 0.91	2.48 ± 0.91	0.583
HDL-C, mmol/L	2.60 ± 1.04	1.04 ± 0.23	1.04 ± 0.24	0.469
Creatinine, μmol/L	73.95 ± 24.95	73.46 ± 21.22	74.40 ± 27.90	0.361
LVEF,%	61.06 ± 8.86	61.03 ± 8.45	61.08 ± 9.23	0.887
**Medicine**				
Beta-blocker	1,821 (78.6)	859 (78.2)	962 (79.0)	0.661
ACEI/ARB	1,293 (55.8)	599 (54.6)	694 (57.0)	0.241
CCB	619 (26.7)	277 (25.2)	342 (28.1)	0.122
Nitrate	1,080 (46.6)	524 (47.7)	556 (45.6)	0.318
Statin	2,183 (94.3)	1,042 (94.9)	1,141 (93.7)	0.207

*SAPT, single-antiplatelet therapy; DAPT, dual-antiplatelet therapy; BMI, body mass index; SBP, systolic blood pressure; DBP, diastolic blood pressure; TG, triglyceride; TC, total cholesterol; LDL-C, low-density lipoprotein cholesterol; HDL-C, high-density lipoprotein cholesterol; ACEI, angiotensin-converting enzyme inhibitor; ARB, angiotensin receptor blocker; CCB, calcium channel blockers; LVEF, left ventricular ejection fraction.*

The ultrasonic and transradial access characteristics are shown in Table 2. The ID of the radial artery was 2.57 ± 0.55 mm, which was slightly larger than the outer diameter (OD) of the sheath tube. The mean IMT and PI of the radial artery were 0.26 ± 0.09 mm and 4.42 ± 2.83. During the TRA, the rate of obtaining the right radial artery access was 92.3%. The specific information, such as the average number of attempts, the average arterial access time, sheath retention time, heparin dose, the number of patients with CAD, and hemostasis time, are shown in Table 2. Except for CAD (28.5 vs. 73.7%, *p* < 0.001), there were no statistically significant differences in the indicators that might be related to radial artery injury (internal diameter of the radial artery, retention time of sheath tube, postoperative hemostasis time, and intraoperative heparin dosage).

**TABLE 2:** Preoperative ultrasonic data of radial artery and angiographic characteristics.

	Overall (*n* = 2,316)	SAPT (*n* = 1,098)	DAPT (*n* = 1,218)	*p*-value
**Preoperative ultrasonic data of radial artery**				
ID, mm	2.57 ± 0.55	2.58 ± 0.55	2.56 ± 0.54	0.251
IMT, mm	0.26 ± 0.09	0.26 ± 0.09	0.26 ± 0.08	0.524
PI	4.42 ± 2.83	4.31 ± 2.81	4.52 ± 2.84	0.069
**Angiographic characteristics**				
CAD, n (%)	1,211 (52.3)	313 (28.5)	898 (73.7)	< 0.001
Right radial access site, n(%)	2,138 (92.3)	1,015 (92.4)	1,123 (92.2)	0.828
Attempts	1.32 ± 0.60	1.31 ± 0.60	1.33 ± 0.61	0.459
Arterial access time, min	1.59 ± 0.89	1.58 ± 0.93	1.60 ± 0.86	0.703
Heparin dose, IU/KG	68.67 ± 11.27	68.71 ± 11.19	68.85 ± 11.35	0.768
Sheath retention time, min	26.86 ± 3.50	26.88 ± 3.51	26.83 ± 3.50	0.759
Spasm,%	145 (6.3)	70 (6.4)	75 (6.2)	0.829
Hemostasis time, h	4.52 ± 1.10	4.54 ± 1.14	4.49 ± 1.06	0.26
RAO at 24 h after the TRCA%	144 (6.2)	68 (6.2)	76 (6.2)	0.963

*SAPT, single-antiplatelet therapy; DAPT, dual-antiplatelet therapy; IMT, intima-media thickness; ID, inner diameter; PI, pulsatility index; CAD, coronary artery disease; RAO, radial artery occlusion; TRCA, transradial coronary angiography. CAD was defined as at least 1 vessel with significant stenosis (> 50%).*

### Outcomes: The Rate of Radial Artery Occlusion, Self-Recanalization, and Delayed Occlusion

**FIGURE 2:**
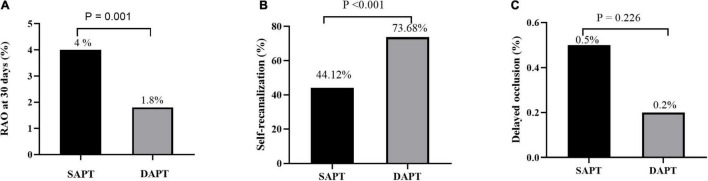
RAO rate at 30 days after the TRCA **(A)**, self-recanalization **(B)**, and delayed RAO **(C)** between the SAPT group and DAPT group. RAO, radial artery occlusion; TRCA, transradial coronary angiography; SAPT, single-antiplatelet therapy; DAPT, dual-antiplatelet therapy.

The rate of RAO at 30 days after TRCA was 2.8%, which was effectively reduced in the dual-antiplatelet group compared with the single-antiplatelet group (4.0 vs. 1.8%, *p* = 0.001). The rate of RAO at 24 h after TRCA was 144/2316 (6.22%), which was evenly distributed among the single/dual-antiplatelet groups (6.2 vs. 6.2%, *p* = 0.963). At 30 days after the operation, an ultrasound revealed that 86/144 patients (59.72%) demonstrated self-recanalization. The self-recanalization rate of patients with RAO at 24 h after the TRCA in the dual-antiplatelet group was (56/76, 73.68%), which was significantly higher than that in the single-antiplatelet group (30/68, 44.12%; *p* < 0.001). Delayed occlusion occurred in 8/2172 patients (0.37%), which was not significantly different between the two groups (0.5 vs. 0.2%, *p* = 0.226). Intuitive information is shown in Figures 2A–C. Unexpectedly, the present study also demonstrated no significant difference in bleeding risk between the single-antiplatelet and dual-antiplatelet groups (Table 3).

**TABLE 3:** Outcomes.

	Overall (*n* = 2,316)	SAPT (*n* = 1,098)	DAPT (*n* = 1,218)	*p*-value
RAO at 30 days after the TRCA,%	66 (2.8)	44 (4.0)	22 (1.8)	0.001
Self-recanalization	86 (59.72)	30 (44.12)	56 (73.68)	0.018
Delayed occlusion	8 (0.3)	6 (0.5)	2 (0.2)	0.226
Symptoms,%	58 (2.5)	26 (2.4)	32 (2.6)	0.69
Hematoma,%	102 (4.4)	51 (4.6)	51 (4.2)	0.592
Perforation,%	9 (0.4)	4 (0.4)	5 (0.4)	1
Arteriovenous fistula,%	2 (0.1)	2 (0.2)	0	0.225
Pseudoaneurysm,%	2 (0.1)	1 (0.1)	1 (0.1)	1
Compartment syndrome,%	0	0	0	–
Spasm,%	145 (6.3)	70 (6.4)	75 (6.2)	0.829
BARC type 2 bleeding%	14 (0.6)	6 (0.5)	8 (0.7)	0.732
BARC type 3 bleeding%	0	0	0	–

*SAPT, single-antiplatelet therapy; DAPT, dual-antiplatelet therapy RAO, radial artery occlusion; TRCA, transradial coronary angiography; BARC, Bleeding Academic Research Consortium.*

### Stratified Analyses of Radial Artery Occlusion

**FIGURE 3:**
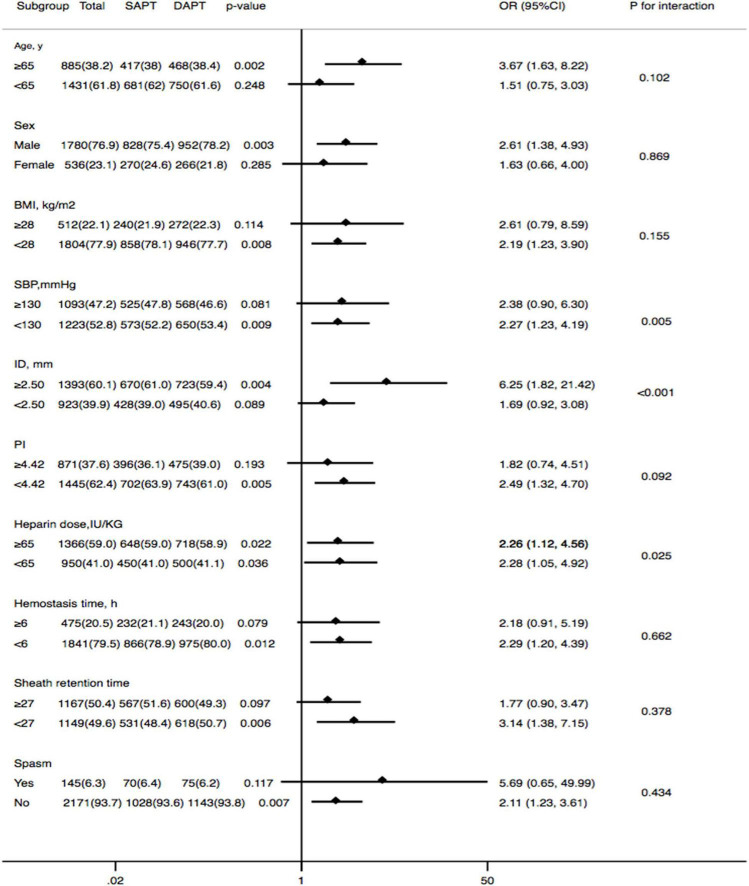
Forest plot investigating the association between DAPT and the prevalence of RAO at 30 days after the TRCA in different subgroups. DAPT, dual-antiplatelet therapy; RAO, radial artery occlusion; BMI, body mass index; SBP, systolic blood pressure; ID, inner diameter; PI, pulsatility index.

The subgroups were classified according to age, sex, BMI, systolic blood pressure (SBP), the ID of the radial artery, PI of the radial artery, heparin dose, hemostasis time, sheath retention time, and spasm, which might affect RAO rates. As shown in Figure 3, the conclusion that dual-antiplatelet therapy was associated with a lower risk of RAO compared to single-antiplatelet therapy persisted in most subgroups. Additionally, there was also no significant interaction between most of the subgroups (except for the subgroups of SBP, ID, and heparin dose).

### Predictors of Radial Artery Occlusion at 30 Days After Transradial Coronary Angiography

**FIGURE 4:**
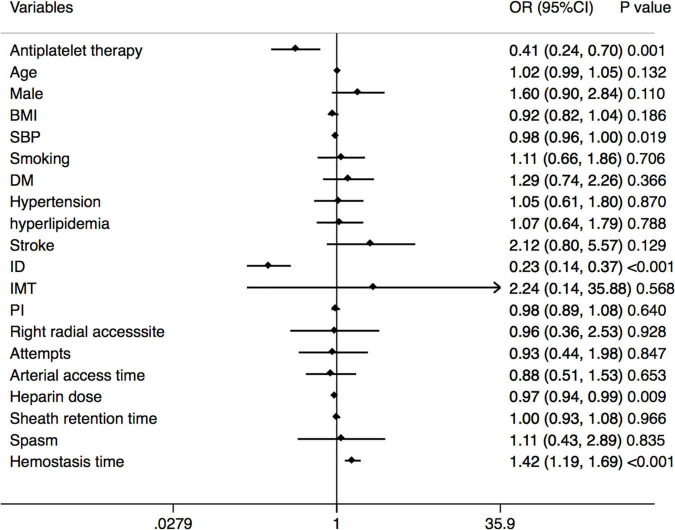
Forest plot of the multivariable logistic regression analysis model exploring the predictors of RAO at 30 days after the TRCA. RAO, radial artery occlusion; TRCA, transradial coronary angiography; BMI, body mass index; SBP, systolic blood pressure; DM, diabetes mellitus; ID, inner diameter; IMT, intima-media thickness; PI, pulsatility index.

A multivariate analysis of RAO at 30 days after the TRCA is shown in Figure 4 to identify the relationship between the antiplatelet strategy and RAO in both directions. Antiplatelet strategy was an independent risk factor for RAO (OR = 0.41, 95% CI = 0.24–0.70, *p* = 0.001). Other risk factors included SBP, ID of the radial artery, hemostasis time, etc.

## Discussion

This study has demonstrated that dual-antiplatelet therapy was associated with a reduced rate of late RAO after the TRCA. Dual-antiplatelet therapy was also linked to self-recanalization of the radial artery, but no benefit has been observed in preventing delayed RAO. In fact, the radial artery was well protected in a safe and relaxed environment using this antithrombotic technique.

### Formation and Urgent Protection of Radial Artery Occlusion

Although RAO rarely causes forearm ischemia in most cases, it can interfere with repeated transradial assessment and may be disastrous for patients requiring CABG and hemodialysis (10, 14). However, the RAO rate remains high; hence, prevention of RAO is of utmost clinical importance (9). The injury process of the radial artery is actually the process of needle puncture and sheath tube insertion. This is a direct physical injury to the intima of the artery, similar to the coronary balloon dilation process. Sheath insertion leads to local endothelial injury, intimal tears, medial dissections, and blood flow cessation in the radial artery, creating a thrombotic milieu, which may also negatively remodel radial artery structure and function (11). Eventually, the formation of a thrombus causes RAO.

### Characteristics of Antiplatelet and Anticoagulant Therapy in Transradial Coronary Angiography Patients

Coronary angiography has a shorter procedure duration, meaning shorter sheath retention time, smaller sheath tube size, and shorter hemostasis time (15), all of which can reduce the occurrence of RAO. Still, RAO incidence during the TRCA was significantly higher compared with PCI. This might be related to the lower amount of heparin and the lack of antiplatelet therapy in patients undergoing coronary angiography. Intraoperative short-term use of heparin could temporarily inhibit thrombi formation and significantly reduce the postoperative short-term RAO rate but had little effect on the long-term RAO rate (16). This conclusion was confirmed in our study. At the 30-day follow-up, the multivariate analysis showed that antiplatelet strategies and heparin dose were associated with late RAO. This conclusion may be a misleading because heparin dosage affects early RAO, and the effect on late RAO might be associated with the backward effect of heparin dosage. In our subdivision, the rate of self-recanalization was significantly higher in the dual-antiplatelet group. A multifactor analysis also showed that dual-antiplatelet therapy was helpful for self-recanalization, but heparin dose was not associated with self-recanalization. This phenomenon is consistent in delayed RAO. Our results were similar to those expected. Long-term postoperative antiplatelet therapy can replace heparin to continue inhibiting thrombi formation.

### Risk Factors Affecting the Late Radial Artery Occlusion

In our study, we paid extra attention to the related factors that might affect the endpoint, such as the patency hemostasis technique and its improvement scheme (17), the model of the sheath tube (15), and the dosage of heparin (18, 19). A previous study has found that the only independent predictor of RAO was the absence of anterograde flow during hemostasis (20). Other studies (21) have also suggested that patent hemostasis, defined as the persistence of antegrade flow during hemostasis, can significantly reduce the RAO rate. However, patency hemostasis requires strict monitoring of blood oxygen saturation and other indicators, significantly complicating postoperative care. Moreover, patency hemostasis cannot be realized in all patients. Previous studies have confirmed that up to 20–50% of patients cannot achieve patency hemostasis (22, 23). The above-described reasons limit the application of patency hemostasis in the real world (24). To simplify the patency hemostasis procedure, Dangoisse et al. (25) have adopted the minimum pressure and the shortest hemostasis time to provide hemostasis and have achieved a 95% patency hemostasis rate. Currently, this method has been applied in multiple centers and achieved the same effect as patency hemostasis (26). Therefore, the above-mentioned hemostatic techniques have also been adopted and have had the same experience. Intraoperative heparin dosage and sheath type between the two groups also showed no significant difference in our study. All of our postoperative radial artery follow-ups were performed by color Doppler ultrasound collecting the parameters of the radial artery to monitor the difference in radial artery ID between the two groups. We found that the ID of the radial artery was only 2.57 ± 0.55 mm, which was significantly smaller than in a French study (27) on adult patients. It is possible that the ID of radial artery is generally small in Asian populations, such as 2.6 ± 0.3 mm in India (28) and 2.5 ± 0.4 mm in Japan (29).

### Dual-Antiplatelet Reduced the Rate of Late Radial Artery Occlusion

Previous studies have emphasized that the application of postoperative antiplatelet drugs is necessary to prevent RAO (30, 31). At the 30-day follow-up on ultrasonography, our results also confirmed that dual-antiplatelet therapy could effectively reduce the RAO rate compared with single-antiplatelet therapy (4.0 vs. 1.8%, *p* = 0.001). All patients with late RAO did not have forearm ischemia and were mainly composed of two parts: Patients with RAO within 24 h postoperatively still without self-recanalization and patients in whom the radial artery was not occluded within 24 h postoperatively but delayed occlusion occurred after 30 days. Thus, dual-antiplatelet therapy plays an important role in promoting self-recanalization of early RAO (4.6 vs. 2.7%, *p* = 0.018), but it does not prevent delayed RAO (0.5 vs. 0.2%, *p* = 0.226). A previous study (28) has suggested that no delayed occlusion occurred once the patency of the radial artery was documented by vascular Doppler ultrasound on the next day after the TRA. However, Sadaka et al. (32) has observed that the incidence of delayed occlusion was 0.9% in his study. Our study also found that the rate of delayed occlusion was 0.37%, which may be associated with chronic remodeling after intima damage of the radial artery and slow microthrombi formation. The multivariate analysis suggested that the ID of the radial artery, PI of the radial artery, and SBP were closely related to self-recanalization and delayed RAO.

In this study, we observed no significant difference in risk of bleeding between the single-antiplatelet and dual-antiplatelet groups. The following reasons may be used to explain this unexpected result. First, the range time of dual-antiplatelet therapy was relatively short, and the follow-up time was not long enough. Additionally, we enrolled patients in this study strictly according to the inclusion/exclusion criteria, which might have led to not including many patients at moderate to high risk of bleeding. Therefore, our conclusions may not be generalized to the patients with high bleeding risk.

## Limitation

This is a prospective observational cohort registration study, and the baseline of the population was uniform. Additionally, this prospective observational cohort study revealed that the strategy of dual-antiplatelet therapy after TRCA was associated with a reduced risk of RAO, but it failed to evaluate the potential causality between dual-antiplatelet therapy and RAO because of the nature of the study. Therefore, future studies are expected to further confirm our findings and investigate the potential causality.

The absence of monitoring all patients to verify the patency hemostasis may be the main subject of regret, but we could only use minimal pressure on the way to achieve patency hemostasis. Our hemostatic regimen is also the closest to the real-world practice of the hemostasis approach. Additionally, we also we also failed to conduct subgroup analysis for patients with high bleeding risk. Therefore, future randomized controlled trials were needed to further confirmed our conclusions in the patients with high bleeding risk.

## Conclusion

Dual-antiplatelet therapy for 1 month after the TRCA was associated with a reduced risk of RAO and deemed safe. However, we should be aware that future randomized controlled trials are required before putting our results into clinical practice because of the limitations of this study.

## Data Availability Statement

The raw data supporting the conclusions of this article will be made available by the authors, without undue reservation.

## Ethics Statement

The studies involving human participants were reviewed and approved by the Clinical Research Ethics Committee of Beijing Tiantan Hospital and Beijing Anzhen Hospital, Capital Medical University. The patients/participants provided their written informed consent to participate in this study.

## Author Contributions

ZQ was responsible for the data analysis and manuscript writing. XY, WC, and JW contributed to the data collection. ZJ reviewed and revised this manuscript carefully. All authors contributed to the article and approved the submitted version.

## Conflict of Interest

The authors declare that the research was conducted in the absence of any commercial or financial relationships that could be construed as a potential conflict of interest.

## Publisher’s Note

All claims expressed in this article are solely those of the authors and do not necessarily represent those of their affiliated organizations, or those of the publisher, the editors and the reviewers. Any product that may be evaluated in this article, or claim that may be made by its manufacturer, is not guaranteed or endorsed by the publisher.
